# Recent Advances and Adaptive Strategies in Image Guidance for Cervical Cancer Radiotherapy

**DOI:** 10.3390/medicina59101735

**Published:** 2023-09-27

**Authors:** Beatrice Anghel, Crenguta Serboiu, Andreea Marinescu, Iulian-Alexandru Taciuc, Florin Bobirca, Anca Daniela Stanescu

**Affiliations:** 1 Faculty of Medicine, “Carol Davila” University of Medicine and Pharmacy, 020021 Bucharest, Romania; beatrice.anghel86@gmail.com (B.A.); alexandertaciuc@gmail.com (I.-A.T.); florin.bobirca@umfcd.ro (F.B.); stanescuancadaniela@yahoo.com (A.D.S.); 2 Department of Radiation Oncology, Sanador Oncology Centre, 010991 Bucharest, Romania; 3 Department of Histology, Carol Davila University of Medicine and Pharmacy, 020021 Bucharest, Romania; 4 Radiology and Imaging Department, Carol Davila University of Medicine and Pharmacy, 020021 Bucharest, Romania; 5 Nuclear Medicine Department, Oncological Institute “Prof. Dr. Alexandru Trestioreanu”, 022328 Bucharest, Romania; 6 General Surgery Department, Cantacuzino Clinical Hospital, 73206 Bucharest, Romania; 7 Department of Obstetrics and Gynecology, St. John Emergency Hospital, Bucur Maternity, 040292 Bucharest, Romania

**Keywords:** cervix cancer, image-guided brachytherapy, ART, external beam radiotherapy, IGRT

## Abstract

The standard of care for locally advanced cervical cancer is external beam radiotherapy (EBRT) with simultaneous chemotherapy followed by an internal radiation boost. New imaging methods such as positron-emission tomography and magnetic resonance imaging have been implemented into daily practice for better tumor delineation in radiotherapy planning. The method of delivering radiation has changed with technical advances in qualitative imaging and treatment delivery. Image-guided radiotherapy (IGRT) plays an important role in minimizing treatment toxicity of pelvic radiation and provides a superior conformality for sparing the organs at risk (OARs) such as bone marrow, bowel, rectum, and bladder. Similarly, three-dimensional image-guided adaptive brachytherapy (3D-IGABT) with computed tomography (CT) or magnetic resonance imaging (MRI) has been reported to improve target coverage and reduce the dose to normal tissues. Brachytherapy is a complementary part of radiotherapy treatment for cervical cancer and, over the past 20 years, 3D-image-based brachytherapy has rapidly evolved and established itself as the gold standard. With new techniques and adaptive treatment in cervical cancer, the concept of personalized medicine is introduced with an enhanced comprehension of the therapeutic index not only in terms of volume (three-dimensional) but during treatment too (four-dimensional). Current data show promising results with integrated IGRT and IGABT in clinical practice and, therefore, better local control and overall survival while reducing treatment-related morbidity. This review gives an overview of the substantial impact that occurred in the progress of image-guided adaptive external beam radiotherapy and brachytherapy.

## 1. Introduction

Cervical cancer is one of the most common malignancies in women worldwide and one of the deadliest forms of cancer with a high burden of disease in developing nations [1,2]. Infection with high-risk subtypes of the human papillomavirus is one of the most important risk factors involved in carcinogenesis [3,4]. Programs and health policies have influenced access to different levels of prevention. The research and development of a vaccine has been proven to significantly reduce the risk of developing cervical cancer in young women [5] but, when diagnosed in advanced stages, the outcomes for cervical cancer remain concerning. The 5-year OS for patients with regional disease is approximately 55% [6,7].

Chemoradiation (CRT) followed by brachytherapy (BT) is the main treatment for locally advanced cervical cancer (LACC) [8]. The addition of concurrent chemotherapy to external beam radiotherapy (EBRT) has improved the prognosis but treatment-related toxicity and distant recurrence remain a challenge [9]. Since 1999, CRT has been recognized as the standard of care in LACC with the results of five randomized controlled phase III trials showing a benefit of 30% to 50% survival advantage by using cisplatin-based chemotherapy to radiation (GOG 85, GOG 120, SWOG 8797/ Intergroup 0107, RTOG 9001) [10,11,12,13,14]. Conventional radiotherapy for cervical cancer is based on cervical examination, 3D conformal radiotherapy, and 2D intracavitary brachytherapy. Definitive CRT for early-stage cervical cancer has shown excellent local control. Compared with 2D or 3D EBRT, intensity-modulated radiation therapy (IMRT) refers to delivering clinical targeted doses using multiple beam angles and field shapes while protecting normal organs such as marrow-containing pelvic bones, bowel, rectum, and bladder [15,16,17,18]. With advances in imaging modalities, 3D-IGABT with CT or MRI has been frequently used to refine target coverage and decrease the dose to normal structures [19].

With this review, we would like to emphasize the role of recent imaging tools such as PET–CT, and MRI introduced in the treatment of LACC and technological advances in IGRT and IGABT.

## 2. Imaging Modalities in Radiotherapy Planning

### 2.1. The Role of MRI

The FIGO staging is driven by clinical examination, proctoscopy, cystoscopy, and colposcopy in relation to imaging. The old system (FIGO 1999, 2009, 2014) was incorrect, with nearly one-third of stages IB-IIIB cancer being under-staged and more than half of the cases with stage IIIB cancer being over-staged [20]. Limitations have been described regarding size, level of involvement for surrounding structure, and nodal status [21]. After pelvic examination and biopsy +/− colposcopy, to obtain a better initial assessment of pelvic tumor extent, a pelvic MRI is mandatory to guide treatment options. MRI is a noninvasive investigation that can determine an accurate estimation of tumor characteristics (size, parametrial, and pelvic sidewall involvement) as well as lymph node status (pelvic and abdominal lymph nodes), with findings pointing to an accurate staging, prognosis, and treatment planning. This imaging study is vital for diagnosis, monitoring, and follow-up [22].

MRI is indispensable for radiotherapy planning for its accuracy to detect soft tissue tumor involvement and to keep tumor regression under surveillance [23]. Residual disease following EBRT is outlined on MRI for adaptive brachytherapy planning [24].

**Figure 1:**
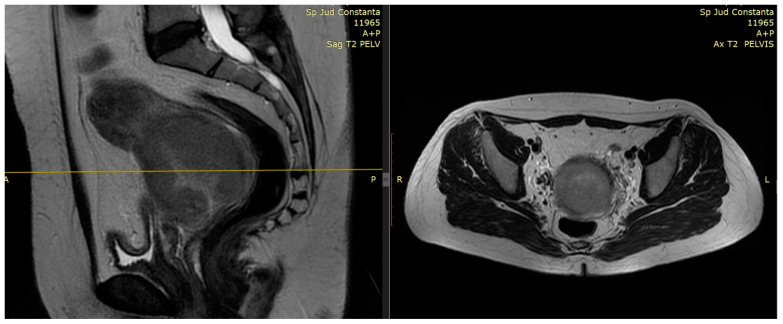
Patient 1 with FIGO stage IIIC2 was treated with EBRT and 3 fractions of HDR CT-based brachytherapy. T2-weighted pretreatment MRI with 6.8 × 6 × 6.2 cm hypointense vaginal wall tumor and superior 1/3 vaginal invasion, bilateral parametrial involvement—sagittal view and axial view.

**Figure 2:**
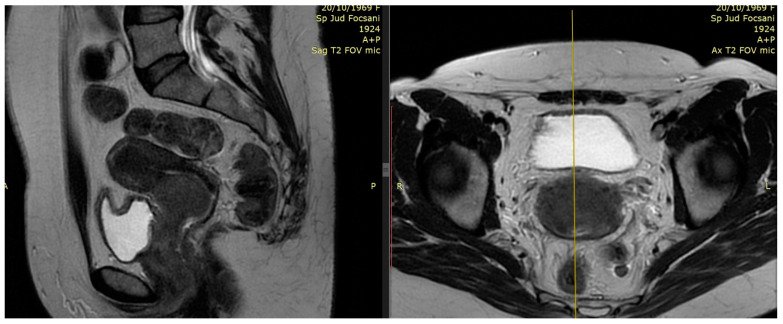
Patient 2 with FIGO stage IVA was treated with EBRT and 3 fractions of HDR CT-based brachytherapy. T2-weighted pretreatment MRI: 4.5 × 5 × 4.6 cm tumor indicating complete vaginal invasion up to urethral meatus and posterior bladder wall involvement—sagittal view and axial view.

T2-weighted provides superior resolution to describe primary tumor and soft tissue invasion and for proper imaging of at least two planes (sagittal, coronal, and axial) with detection of extension into the uterus, parametria, and adjacent organs [25]. For a reliable volumetric definition of the target volume, it is relevant to visualize the cervical tumor in multiple planes. An example of collaborative work with the MRI department and Radiotherapy department, for the best interest of our patients, is reviewed in Figure 1 and Figure 2.

In the past, the treatment plan for cervical cancer was developed based on bony landmarks using 2D planning. Analysis of previous studies with MR imaging has looked at tumor coverage with standard pelvic fields and alignment to bony anatomy resulting in geographical miss in up to 66%. MRI is crucial to ensure that the target is satisfactorily covered. When planning the treatment, an MRI should be conducted in the same position as the planned treatment with two options: MR simulation or image registration to a CT simulation. Images should be obtained with 1.5–3 T scanners with body coils. To prevent peristaltic motion, glucagon could be given intravenously prior to or during the exam. To define better, target and OARs multiplanar T2-weighted images are helpful. Improved reproducibility has been noted among experts, particularly in delimitating the parametrium once guidelines are published for delineation targets and OARs on T2-weighted MRI. With this information, MRI in a treatment position represents a standard imaging procedure for 3D conformal and IMRT planning. The large inter- and intrafraction anatomic changes of pelvic organs have an impact on both target volume coverage and dose to normal OARs. This is a factor that strongly influences EBRT precision and considerable motion can be observed between fractions due to bladder filling, rectal filling, and internal motion [26]. Great results have been achieved with the availability of MRI in brachytherapy and superior visualization of the residual tumor dimensions at the time of brachytherapy together with developments of new applicators with interstitial needles to cover laterally the parametrial involvement that would have been underdosed with classical applicator models [27].

**Figure 3:**
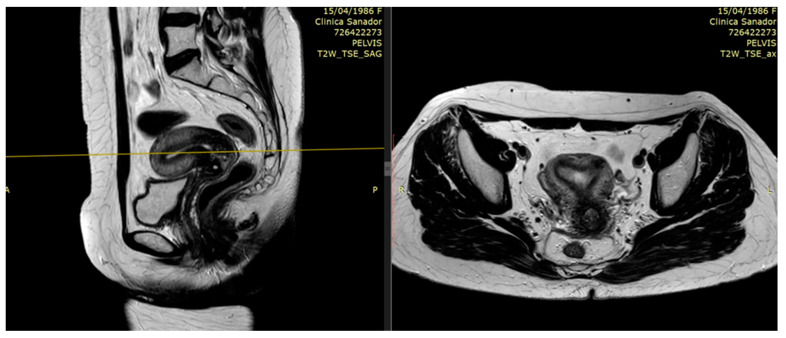
Patient 1 T2-weighted pre-brachytherapy MRI (5th week of CRT): 1.7 × 1.7 × 2.8 cm residual posterior cervix tumor and irregular tumor signal extending to left parametrium (parametrial invasion)—sagittal view and axial view.

**Figure 4:**
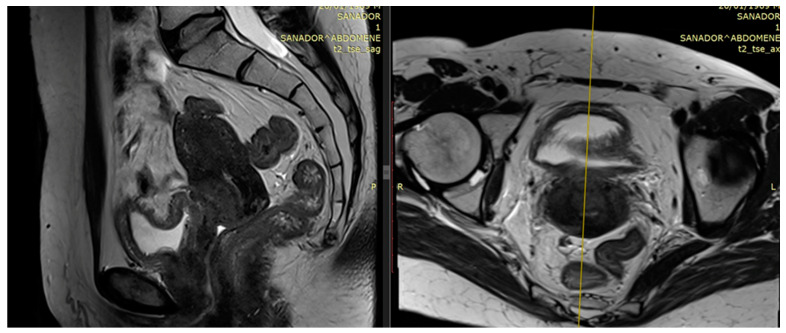
Patient 2 T2-weighted pre-brachytherapy MRI (5th week of CRT): residual tumor and irregular tumor signal extending to parametrium, bladder, and vaginal wall—sagittal view and axial view.

Examples of residual large tumors diagnosed and treated in our institutions with MRI performed for planning brachytherapy are available in Figure 3 and Figure 4.

MRI has excellent accuracy to determine remaining tumor early after radiation therapy as well as disease recurrence during follow-up imaging [28]. An important challenge is to identify tumor recurrence versus post-therapy sequelae on MRI (treatment-related fibrosis, inflammation, and necrosis) in view of reducing the high false positive rates when MRI is performed within 3–8 weeks post-treatment. Correctly identifying tumor recurrence is necessary for more than 6 months between treatment and post-therapy assessment [29]. With functional MRI, such as diffusion-weighted imaging (DWI), a potential noninvasive biomarker for tumors, it has been observed that the tumor shrinks with treatment, and water mobility increases. Another indicator of tumor response that may increase and be reliable is the apparent diffusion coefficient (ADC). In more recent data, DWI-MRI is considered an early biomarker of tumor response to chemoradiation with promising results in preliminary studies, and further work is needed in this direction [30]. The availability of MRI in the diagnostic area has brought accurate information for our patients, although, due to the lack of preplanning for infrastructure and qualified personnel, it is hard to implement in radiotherapy departments after department development.

### 2.2. The Role of PET–CT

In cervical cancer, one of the unfavorable prognostic factors is the presence of lymph node (LN) metastasis. Compared to CT and MRI, FDG PET is more reliable for detecting pelvic and paraaortic (PA) LN metastasis [31,32]. At diagnosis, a study showed that FDG PET detected nearly half of the patients (47%) with positive LN metastasis [33]. In PET-positive LN scans, all had pelvic nodes, 35% PA, and 12% supraclavicular LN metastasis. LN detection via PET–CT could upstage the clinical staging and, therefore, modify the treatment decision and integrate the extended field for the inclusion of metastatic LN into the radiotherapy volume. Excellent local control and acceptable morbidity are offered with IMRT when the PA region is included in the radiotherapy plan [34].

In a randomized trial, 129 patients (I-IVA stages) with positive and negative PA LNs on MRI staging were randomized to have additional FDG–PET for staging or not. Patients with PA LNs FDG avid had an extended field to include LN metastasis. Seven patients had extra pelvic metastases on the PET scan, mainly paraaortic LNs. The conclusions of the study reveal the importance of two imaging modalities, such as pretreatment FDG–PET together with MRI, which can improve the detection of extra pelvic metastasis, particularly PA LNs, and facilitate the selection of cases with extended-field RT. Adding FDG–PET into the staging process has not provided a survival advantage, although the number of PA LN relapses was lower [35]. On the other hand, a more recent study by Su et al. suggests that pretreatment [18] FDG PET–CT might be linked with longer survival in patients with stage IB-IVA treated with CRT, especially in the IGRT and IGABT era [36].

A French analysis highlights the existence of false negative results in 12% of patients staged with negative PA involvement on PET–CT and found positive on laparoscopic PA lymphadenectomy [37]. There is a debate about exposing patients to potential surgical morbidity for those with a negative PET scan. There is a real potential for PET–CT to be integrated into treatment planning to increase the dose to positive LNs with a simultaneous integrated boost (SIB) technique with a perspective to improve regional control [38].

The most recent ESGO/ESTRO/ESP guidelines for the management of patients with cervical cancer—update 2023 Davida Cibula et al.—recommend that nodal status in LACC (T1b3 and higher—except T2a1) and in early-stage disease with highly suspicious LN on imaging should be investigated with a positron emission tomography–computed tomography (PET–CT) [39].

2-deoxy-2 [^18^ F] fluor-D-glucose PET–CT appears to be the best imaging modality for all stages of lymph node involvement with sensitivity and specificity of 82% and 95%, respectively [40,41], and is generally being used for radiation treatment planning in LACC. PA region is essential to be assessed, as 4–30% of patients with LACC have LN metastasis at this level, while 11% have local nodal failure after CRT, from which almost 70% are in PA LNs, as shown by the retroEMBRACE study [41,42].

**Figure 5:**
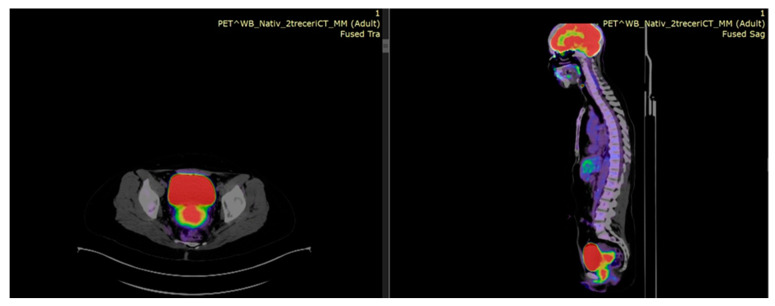
An example of pretreatment [^18^F] FDG PET–CT showing a locally advanced cervical tumor with complete vaginal invasion and bladder involvement up to urethral meatus—axial view and sagittal view.

**Figure 6:**
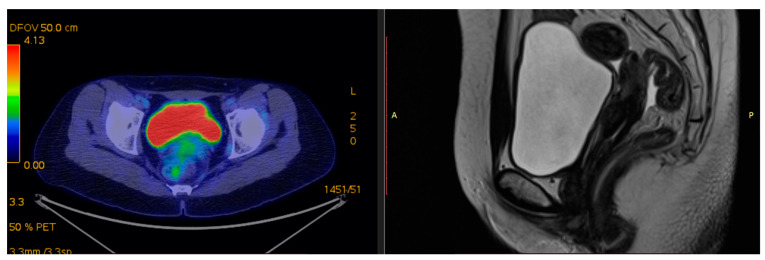
Post IGRT/CRT and IGABT treatment modalities in a FIGO IIIC1 case—left (axial view)—^18^FDG PET–CT low FDG uptake (SUV max 4) and right (sagittal view)—T2-weighted MR images—no mass in the cervix with low cervical signal intensity. Colposcopy with biopsy was negative for malignancy.

PET–CT is an important diagnostic tool in LACC management and should be incorporated into the planning process to appreciate and assess the risk for lymph nodes and distant metastasis. In Figure 5 and Figure 6, we provide images with the current role of PET–CT from our database for two different settings: pretreatment work-up and post-treatment follow-up for patients treated with EBRT and image-guided brachytherapy.

## 3. Advances in External Beam Radiotherapy

### 3.1. From 2D to 3D Conformal Radiotherapy

In the past, the treatment of LACC with RT has been implemented with 2D EBRT using anatomical landmarks on X-rays to be able to encompass the primary disease and the potential spread. Two parallel opposed fields (AP–PA) were the basic techniques of EBRT. Later, the “four-field box” technique was introduced to achieve better conformality with four large treatment fields, with the upper border at the level of the aortic bifurcation (L3–L5 vertebral body levels; T12 level in case of extended field in case of paraaortic nodes being involved), the anterior border placed anteriorly to the pubic symphysis, and another border posteriorly outlining the sacrum at the S3/S4 level.

The lateral borders are 1.5–2cm lateral to the pelvic brim and the inferior border is the bottom of the obturator foramen. Although easy to implement in practice, geographical misses have led to reduced LC [43]. CT invention was brought into RT planning after the 1990s, marking the evolution from 2D to 3D conformal RT. Based on the information obtained from a CT scan of the patient imaged in the same position for treatment and in a reproducible way has helped clinicians, physicists, and radiation therapists to understand concepts related to target delineation—gross tumor volume (GTV), clinical target volume (CTV), and planning target volume (PTV)—published in ICRU Report 50 and ICRU Report 62 [44,45]. This technique utilizes anatomical landmarks to better shape the dose distribution of the PTV with the protection of OARs with multileaf collimators (MLCs) [46]. Three-dimensional conformal RT provides volumetric dosimetry and has the advantage of quantifying and correlating treatment outcomes and toxicities.

### 3.2. Intensity-Modulated Radiotherapy (IMRT) Volumetric Intensity-Modulated Arcs (VMAT)

IMRT is a radiotherapy technique that warrants rigorous conformation of the radiation dose to the target volume. Through IMRT, there is potential to significantly reduce long-term morbidity and improve local control. Compared with conventional EBRT, IMRT uses small beamlets with variable intensity and better conformality to 3D target volumes while minimizing the dose to critical adjacent structures. In 2002, excellent results have been reported with the initial implementation of IMRT for women with gynecological cancers.

TIME-C, Uterus-11, PARCER, INTERTECC-2, Huang et al., and Ghandi et al. are numerous major studies involving advances in radiation therapy techniques with significant lower bowel and bladder toxicity [47,48,49,50]. Emerging evidence has shifted the balance increasingly in favor of the ordinary use of IMRT. IMRT can be given via multiple static fields or in arcs, a newer radiation technique known as volumetric intensity-modulated arc radiation therapy (VMAT). Conformality, faster treatment time, and fewer monitor units are some of the main advantages offered by VMAT.

IGRT can deliver a higher dose to the primary tumor and areas at high risk for recurrence with the SIB technique without exposing adjacent normal organs to radiation.

IMRT on a routine basis faces issues such as organ movement during RT (intrafraction) and during treatment (interfraction). Jadon et al. [51] reported that organ motion in EBRT for cervical cancer is present and the degree of movement during radiation therapy should be considered. Bladder and rectal filling influence uterine and cervical motion [52].

As pelvic organ motion seems to be patient-specific, personalized PTV margins and adaptive image-guided radiotherapy (IGRT) have been proposed to cover accurately the target volume while enlarging the normal tissues’ protection. As IMRT planning can spare normal OARs, IMRT plans and SIB of LNs are a great strategy to reduce radiation-induced normal tissue complication probability while improving outcomes in a shorter overall treatment time.

### 3.3. Adaptive External Beam Radiotherapy

IGRT is a dynamic complex process of performing imaging before daily radiotherapy with the intent of achieving target accuracy and precision by correcting geometric and anatomic variations. IGRT techniques consist of planar or volumetric imaging to obtain tighter treatment margins. Highly conformal techniques such as IMRT and stereotactic body radiotherapy (SBRT) demand a high level of setup reproducibility to ensure that the planned dose is delivered to the interest area. Planar IGRT techniques compare 2D radiographs in the same treatment position with digitally reconstructed radiographs (DRR), images obtained from simulation. This allows accurate matching of the radiograph from simulation on bony anatomy with the radiograph from treatment. Volumetric IGRT techniques provide 3D imaging comparable with the initial simulation imaging to check position. This allows for soft tissue and bony anatomy as well (volumetric IGRT: cone-beam CT, CT on rails, and megavoltage CT imaging) [53].

IGRT plays a central role in modern radiotherapy, especially for hypofractionation and stereotactic treatments. It can boost confidence that radiotherapy treatment is in the desired size and margins can be tailored when appropriate. Many factors can determine anatomic deformations to the tumor and OARs throughout a course of treatment:

Firstly, anatomic motion is caused by the system (e.g., musculoskeletal, gastrointestinal, genito-urinary, cardiac, and respiratory).

Secondly, the treatment-induced changes such as tumor reduction, regrowth resulting from accelerated repopulation, weight gain or loss, concomitant chemotherapy, and fibrosis of normal structures.

It can happen at three levels: offline between sessions, online immediately prior to treatment, and real-time during radiotherapy. Based on a predetermined set of scenarios, simple forms of adaptive radiotherapy apply correct measures and will define the concept of a multiadaptive image-guided radiation therapy (IGRT) plan.

In LACC, the uterus and cervix change position during treatment delivery due to variations in rectal and bladder filling and tumor shrinkage during RT [53]. Deformable image registration for transferring anatomic contours and dose between images are described in recent practice advances for forms of adaptive radiotherapy and software tools to analyze automatic treatment planning and deformable dose summation [54].

**Figure 7:**
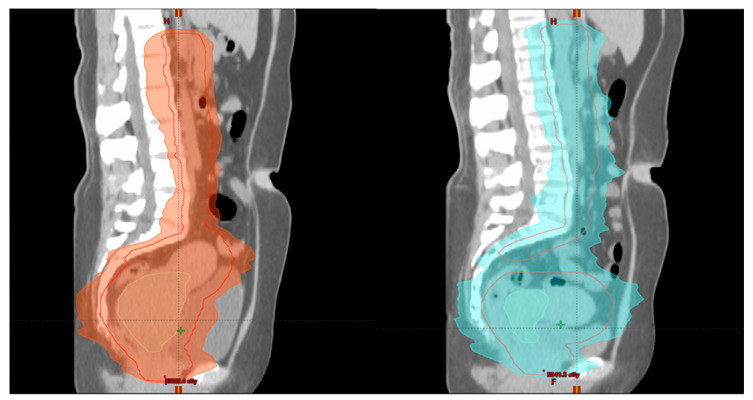
Patient 1 with FIGO stage IIIC2 LACC: **left** image (week 1)—initial plan of EBRT and **right** image (week 3) during EBRT—adaptive planning (sagittal view) for tumor shrinkage.

**Figure 8:**
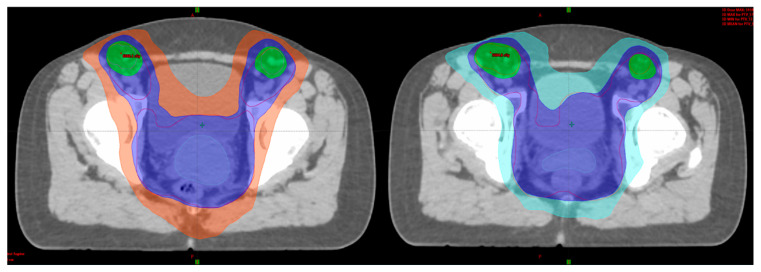
Patient 1 with FIGO stage IIIC2 LACC: **left** image (week 1)—initial plan of EBRT and **right** image (week 3) during EBRT—adaptive planning (axial view) for tumor shrinkage.

Adaptive radiotherapy is defined as a temporal adjustment of the treatment plan delivery to a patient, according to objective anatomic changes caused by weight loss, tumor shrinkage, or internal motion (Figure 7 and Figure 8).

A new concept of internal target volume (ITV) is generated to account for various treatment positions for LACC by performing a simulation with a full and an empty bladder and then combining the CTV to be taken into consideration for every move between these two bladder filling extremes [55]. A margin between 3 and 7 mm (PTV) is added to the ITV to fully encompass setup and position errors. Then, volumetric IGRT is applied with cone beam CT (CBCT) to verify the position of the CTV and PTV daily prior to RT delivery. More advanced adaptive strategies have made space for a highly advanced work for treatment delivery called “plan of the day” or “online adaptive RT”. These approaches include same-day replanning and recently published review articles are available in the literature [56].

In special scenarios, after paraaortic exploration or hernia repairs, open or laparoscopic, it is vital to use IGRT techniques to protect the wound and prevent complications or even delays in starting radiotherapy for LACC [57].

## 4. Advances in Brachytherapy

### 4.1. From 2D Brachytherapy (2D-BT) to 3D Image-Guided Adaptive Brachytherapy (3D-IGABT)

Brachytherapy is an essential component of the treatment of LACC in addition to concurrent CRT. Internal radiation therapy allows an important dose escalation to the gross tumor with the advantage of rapid dose fall-off. This dosimetry analysis shows effective dose coverage of the target while protecting the OARs through technical adjustments of implants (hybrid implants with intracavitary and interstitial needles) and multi-parametric 3D treatment planning [58]. Intracavitary brachytherapy (IC BT) can be accomplished using low dose rate (LDR), pulsed dose rate (PDR), or high dose rate (HDR) sources. HDR has the advantage of exposing the patient care team to less radiation than LDR and there is a need for fewer logistic measures dedicated to source storage and fewer radiation safety measures compared to LDR.

There is an increased risk of exposure, for example, by selecting the inappropriate source strength when retrieving source capsules from storage or incorrect order when implanting sources. For these reasons, IC BT has changed to HDR delivery using remote after-loaders. HDR with a treatment planning process by a computer could optimize the dose to target critical structures. HDR treatments are evaluated using conversion to radio-biologically equivalent doses (EQD2) integrated into clinical practice with caution that comes with model-based conversion (see American Brachytherapy Society Excel worksheet to convert HDR doses to 2Gy equivalent doses). The Manchester system, first introduced in 1938 and then modified in 1953, has established the brachytherapy dose prescription to a specific point; point A was defined as a point 2 cm lateral to the center of the uterine canal and 2 cm superior to the mucosa of the lateral fornix. Later, this system was modified to be relative to the brachytherapy applicator itself to be better visualized on X-ray, which was defined as 2 cm superior to the external cervical ostium and 2 cm lateral to the tandem portion of the applicator [59,60]. Personalization of treatment planning and target-based dose prescription are concepts described in modern BT.

Several options are available: ring and tandem or tandem and ovoid devices, both with the possibility of increasing the complexity of the implant, from an IC one to a hybrid approach (IC plus interstitial needles). The rationale for this mixed approach depends on tumor location, size, and degree of vaginal extent. IC device is recommended for small tumors (<3 cm), with minimal extension into the vagina and no parametrial disease.

When a tumor is large, irregular in shape, and has parametrial involvement, then a hybrid IC/IS can be a better solution with superior dosimetry. In the scenario where a tumor is very large, with extensive vaginal and parametrial involvement and sometimes adjacent organ involvement (e.g., bladder or rectum), interstitial therapy is recommended. For IGABT treatment planning, MRI or CT can be used with significant advantages over point-based planning and there are consensus guidelines for IGBT target delineation [61].

MRI near the time of brachytherapy is an asset and can be used for the identification of GTV. The dose prescribed is to the contouring volume formed by high-risk CTV, which includes GTV, the entire cervix, and macroscopic extension or parametrial involvement. After 45 Gy in 25 treatments to the pelvis (common dose), fractionation schemes to achieve more than 80 Gy Eqd2, including 7 Gy × 4 fractions, 8 Gy × 3 fractions, and 5.5–6 Gy × 5 fractions. Numerous institutional series have showed great results with limited toxicity. In 2008, a prospective observational study was initiated, called EMBRACE, to analyze outcomes from the application of MRI-based IGBT in a multicenter, international population according to Gynecological Groupe Européen de Curiethérapie and the European Society for Radiotherapy & Oncology (Gyn GEC—ESTRO) standards [62,63].

As we know, numerous studies show that IMRT/3D–IGABT is linked to improved survival and reduced gastrointestinal and genitourinary toxicity in patients with LACC compared with those who received 2D EBRT/BT [64,65]. The RetroEMBRACE cohort, an international observational cohort, enrolled 852 patients treated with IGBT prior to participation in EMBRACE [66].

Institutions’ MRI-based IGRT users apply GEC—ESTRO recommendations [62] and institutions using CT IGRT are instructed to contour only high-risk CTV [66]. The MRI-based IGBT cohort has a 95% local control at 3 years for < 5 cm tumors, while 85% for tumors > 5 cm. The 3- and 5-year OS rates were 74% and 65%; it is notable that 70% of the overall deaths were due to recurrent disease. Within the Embrace I study, excellent outcomes have been reported, with 51 months being the median follow-up and actuarial 5-year local control was 92% (95% CI 90–93%), with similar local control across all FIGO stages [67]. Local failures in lymph nodes or distant failures occur about half of the time and most local failures have been exposed within the high-risk CTV or intermediate-risk CTV, correlating a dose-dependent relationship [68].

The primary target dosimetry goal was an EQD2 of 85 Gy, assuming an α/β ratio of 10. EMBRACE I early results support the use of combined IC/IS BT, particularly for larger tumors, to reach the target dose with an acceptable toxicity profile [69].

Results from a cancer center in Romania with high addressability of patients with cervical cancer discuss the need to implement the hybrid IC/IS IGABT approach to fulfill the requirements for dose targets and respect surrounding normal tissue constraints [70]. A prospective French STIC trial [71] randomized patients between 2D and 3D BT planning and showed that 3D IGABT is recommended, effective, and safe, with better local control and grade 3–4 toxicity reduced to half, favoring IGABT. In the EMBRACE II cohort, dosimetry and planning advanced features were implemented for patients with FIGO IB-IVA disease with EBRT and IG-IMRT to 45 Gy/25 treatments with MRI-based IGABT with concurrent cisplatin.

**Figure 9:**
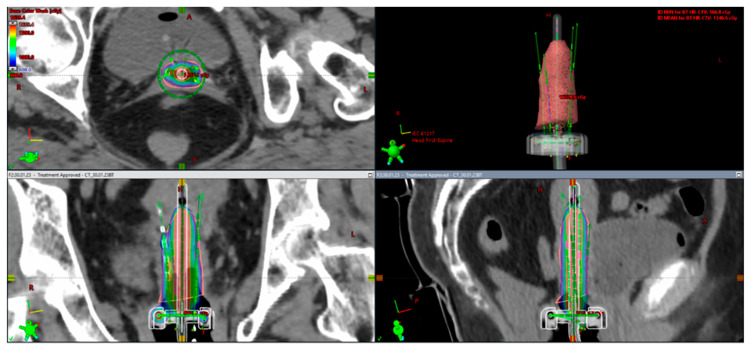
Combined approach (IC + IS). In this example of FIGO IIIC2, a large cervical tumor at presentation with bilateral parametrial involvement and lower uterine segment infiltration and partial response to treatment pre-brachytherapy with residual tumor to the inner third of parametria bilaterally led to the decision to choose a combined approach (implant representation ring and uterine tandem with 4 needles).

**Figure 10:**
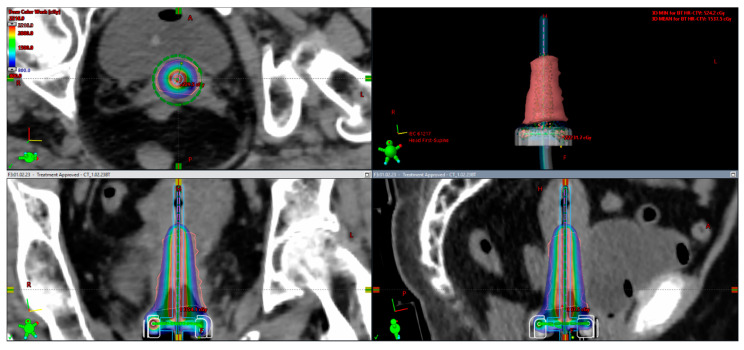
IC approach. The rationale for intracavitary application for the same patient as described in Figure 9: for the last procedure, due to poor blood results (severe thrombocytopenia), the decision was made for an intracavitary approach in order not to prolong overall treatment time (implant representation ring and uterine tandem).

Figure 9 and Figure 10 show sample images from a patient treated with a combined IC/IS approach (Figure 9) and then with an intracavitary BT technique (Figure 10) with CT-based image guidance. This is an image-based HDR BT plan for a 64-year-old woman with FIGO IIIC2 cervical cancer (FIGO 2018). After EBRT with concurrent cisplatin, the plan was to receive a brachytherapy boost of 24 Gy in three fractions. She underwent first and second fractions with a combined technique (Figure 9), consisting of a ring and tandem applicator supplemented by the placement of four interstitial needles. The legend shows the isodose curves (colored lines) with a dose-expressed color wash. Not to prolong her OTT while recovering from her low platelet count, she received her last treatment, an IC BT implant (Figure 10).

### 4.2. Adaptive Brachytherapy

The initial implementation of BT in cervical cancer has started with point A dose prescription 2D X-ray dose planning together with standard dose distributions. Unfortunately, point A is a poor substitute for evaluating the dose to a 4-dimensional (4D) target. GEC–ESTRO recommendations introduced image-based target assessment and possibilities to improve prescription, optimization, and dose reporting in a reproducible way. IGABT is conducted in the last 2–3 weeks of an overall treatment time of 7 weeks (50–56 days considered a time constraint) to make the most of the advantage of tumor regression. Tumor regression is more limited in the last weeks, and it may lead to underestimated tumor dose in case the same dose planning concept is applied for all fractions of BT because of the potential reduction of tumors in the high-risk volume. The concepts of “high risk” CTV and “intermediate risk” CTV are implemented in modern BT and are residual gross disease at BT time and, respectively, potential microscopic residual disease. For each BT fraction, there is potential through image-guided dose optimization to spare OARs by tailoring the dose to the target. Significant OAR movement has been observed and it has been highlighted in dosimetry analysis to adjust a repeat adaptive planning for each BT treatment [72,73].

The report released in 2018 by the EMBRACE II study reviewed the outcome and prospect of two decades of progress within the GEC—ESTRO Gyn working group. MRI-guided adaptive BT with combined IC/IS techniques is incorporated within the study and specific dose-volume constraints are recommended for adaptive targets and OARs, for EBRT image guidance for specific targets and techniques (IMRT, IGRT, and SIB for nodal disease), and concurrent CRT. The new concept described is IGABT for its precise delivery of the dose target and excellent protection of OARs. Taking into consideration the time-consuming procedure and the resources needed, IGABT contributes to a reduction in OTT by increasing the fraction size. Another measure adopted is to finish the treatment of EBRT and BT in 7 weeks [74,75].

MRI is the preferred image modality and gold standard for superior soft tissue contrast. The concept of IGABT is based on two main topics: imaging at the time of BT with the applicator in situ and then the same dose planning on the anatomy as the dose delivered.

**Figure 11:**
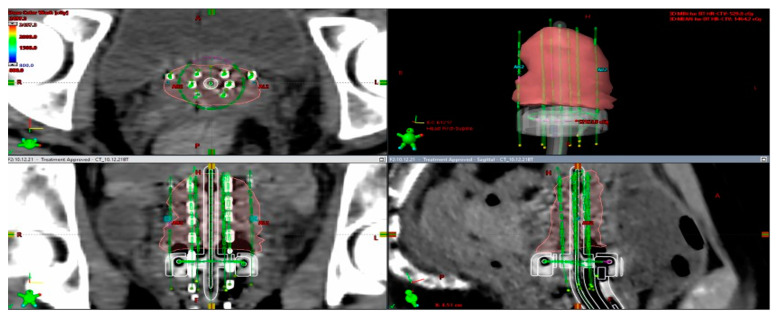
Rationale for interstitial application. In this example, bilateral parametrial involvement at the time of diagnosis and residual tumor to the inner third of parametria bilaterally led to a decision to choose a hybrid approach (implant representation ring and uterine tandem with 8 needles).

An adaptive approach results in two major steps: (1) the right applicator to the anatomy and (2) 3D dose optimization with the advantage of having the applicator in situ. Inserting the suitable applicator results in a direct relation between the radioactive source and the topography. For example, in a small tumor, IC BT could be the right choice but, in large tumors with poor response, IC applicator and interstitial component are suitable [76,77]. Another fundamental change with IGABT is the improvement in dose for high-risk CTV, especially for large tumors, with suboptimal dose coverage by 85 Gy EQD2 with standard nonoptimized IC BT (Figure 10 and Figure 11). 

3D-IGABT compared with 2D-BT techniques shows a superior safety profile and efficacy in terms of LC. The up-to-date standard for BT is MR image guidance. The major advantage is the dose conformality given by BT regarding both the volume (3D) and the time (4D) [78]. Before each implant, repetitive imaging gives the possibility to adapt the dose to the tumor volume in regression.

A recent systematic review and meta-analysis by Kim et al. have highlighted the important data in the EMBRACE II study stating that 3D-IGABT is a cost-effective option supporting routine use in the treatment of LACC [79,80,81,82]. A multidisciplinary team is involved and includes a radiation oncologist, physicists, anesthesiologist, radiation therapists, and nurses. This holistic approach can integrate artificial intelligence (AI) systems to develop algorithms to perform a safe and efficient procedure. Due to current developments, it is important to bring AI as a possible guardship against intraoperative anaphylaxis [83].

Based on the large application of 3D-IGABT for cervical cancer, the ICRU/GEC—ESTRO launched Report 89, with a focus on adaptive brachytherapy. The concept of 4D (four-dimensional) image-guided adaptive brachytherapy (4D-IGABT) is a new approach with great clinical results and has included the 4D concept, which is formed by three spatial dimensions and time. The four-dimensional IGABT design improves the efficacy-to-toxicity ratio by exploiting the tumor-volume reduction commonly seen in cervical cancer after the first part of treatment. Numerous factors should be considered in real-life practice, such as tumor regression, internal organ motion, and organ filling. The main objective of this concept is a more precise treatment through adaptive contouring, imaging, and replanning, replacement of the applicator, and the addition of the interstitial needles while quality assurance procedures are checked simultaneously.

**Figure 12:**
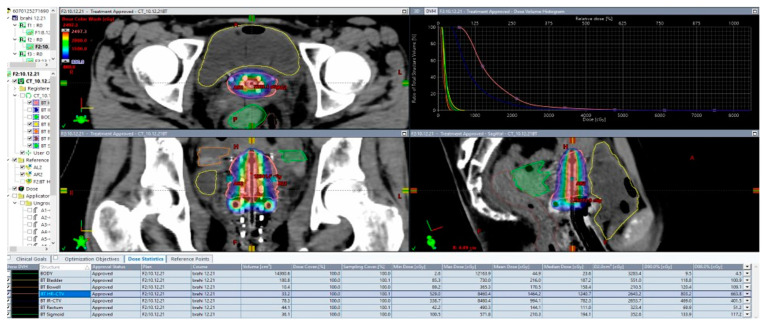
Implant representation ring and uterine tandem with 8 needles with isodose curves–tumor coverage and OARs representation.

In Figure 11 and Figure 12, we would like to emphasize our work on an HDR CT-based BT plan for a 14-year-old girl with FIGO IIIC2 cervical cancer (FIGO 2018). After EBRT with simultaneous cisplatin, she underwent a brachytherapy boost of 24 Gy in three fractions with a combined technique (Figure 11), consisting of a ring and tandem applicator supplemented by the placement of eight interstitial needles. The legend shows the isodose curves (colored lines) with dose-expressed color wash (see Figure 12).

## 5. New Modalities of Radiation Techniques

### 5.1. MRI-LINAC Using Adaptive Radiotherapy

The MR-LINAC is a ground-breaking system with an extensive care path that has the potential to revolutionize cancer treatment, refine treatment, and enable radiotherapy for hard-to-treat cancers [84]. Magnetic resonance imaging-guided radiotherapy (MRgRT) outlines the greatest potential to achieve the therapeutic gains of image-guided delivery of planned radiation dose. Major advances are noticed with the agility of the MRI LINAC to capture tumors and OARs with on-table MRI, to ensure motion management and delivery of the dose prescribed in real-time, and then to adapt the plan on the same day of the treatment while the patient is on the machine table [85,86]. Imaging for therapy guidance, real-time imaging, and gating for intrafractional management are the main topics analyzed. A brand-new concept is adaptive treatment planning for interfractional management.

This increased eligibility for RT or opportunity for reirradiation leads to improved quality of life for more patients. Dynamic real-time adaptation provides the ability to tailor each MRI-guided radiation therapy based on anatomical changes in size, shape, and position of the tumor or near organs. Diagnostic quality MRI with precise radiation delivery allows treatment dose to be escalated and the tumor to be visualized at every moment, leading to increased protection of normal organs, and providing opportunities for better outcomes and fewer side effects [87].

### 5.2. 3D Printing in Cervical Cancer Brachytherapy

To perform brachytherapy, an applicator is needed, a medical device that decreases the radiation received by medical personnel. Interstitial brachytherapy can utilize a transperineal template, which has several hollows, allowing guiding tubes to be inserted directly into the tumor or nearby. After-loading and interstitial brachytherapy are appropriate for cervical cancer due to anatomy and the location in the proximity of the cervical tumor. The basic concept remains to deliver a high dose to the cervical target tumor area while protecting OARs. CT and MRI are frequent imaging tools in cervical cancer brachytherapy and significant progress has been made with technology, software, and image guidance. 3D image guidance in cervical cancer brachytherapy can improve local control and minimize radiation effects in OARs. However, 3D-IGABT needs to be implemented by an experienced physician to manipulate the right applicator, insert it at the right position, and revisit all different patient tumor characteristic scenarios, such as size and location. Occasionally, it can be quite difficult to achieve individual treatment by conventional after-loading or interstitial brachytherapy.

Recent gynecological brachytherapy groups are offering high expectations with the application of 3D printing technology in order to solve this issue and ensure personalized treatment with the potential to improve survival rates. With respect to accurate radiotherapy, personalized RT products can be created for each patient by using 3D printing technology. Personalized products can be designed using this technology to perform preoperative planning. The major objective would be to display tumor shape in an anatomic relationship between the tumor and surrounding structures. The next step will be manufacturing a personalized guidance template. Getting the right optimization from needle positions will translate into favorable target dose distributions. Current advances are upgrading the common applicator to an individual design applicator to fit the patient’s anatomy and achieve optimal accuracy in dose distribution. A few challenges are described in Huo et al.’s review: 3D-printing materials, 3D-printing model construction, and dose distribution calculation [88,89]. The application of a 3D-printing individualized applicator and template could have enormous potential to improve accuracy in brachytherapy in clinical practice.

## 6. Future Directions

Based on clinical evidence and guidelines in the literature, real data are available for clinical implementation in gynecological cancers in the era of pelvic IGRT for routine practice. General recommendations are made for the implementation of an institutional pelvic IGRT protocol. Specific features include consistency between treatment intent and the IGRT approach with consideration for minimum national and international IGRT guidance when daily volumetric IGRT is applied. It is important that appointed appropriate health professionals (RTTs) lead on undertaking IGRT with continuous professional development. An IGRT workflow procedure must be clear and, when difficult cases are encountered, the process can be escalated with transparency and strategic directions. Before implementing advanced adaptive strategies, it is mandatory to ensure a robust IGRT service is already in place [90].

Overall, IGABT has significant improvements in local control for all stages in LACC, particularly with 3D and 4D adaptive measures. However, there is potential for further advances in IGABT with hybrid approaches at the time of BT. Unfortunately, cervical cancer is diagnosed in large numbers and with a significant burden of disease in low- and middle-income countries. The need for brachytherapy equipment, imaging modalities, access to treatment, and state-of-the-art options remains a problem and different approaches and workflows must be adopted for these patients. A few ongoing studies mention the use of concurrent CRT plus BT in combination with immune checkpoint inhibitors in patients with LACC, but limited data are available on the sequence of treatments and overall efficacy and safety.

There are promising ways to refine adaptive radiotherapy in both limited and LACC, tailoring the treatment to the individual patient, tumor burden, and response. Furthermore, it is known that there are important changes in the tumor microenvironment and biological factors are investigated for response to radiation. Radiomics has shown promise in precision medicine and advances are reported that will become a valuable tool for the clinical arena. Additional progress in this direction will offer a possibility to provide, in the future, an assessment of tumor characteristics with personalized biological dose planning and dose painting. Cone beam CT quality and the integration of MR-guided EBRT and BT will need technological developments.

## 7. Conclusions

Imaging modalities are rapidly integrated into current practice in modern radiotherapy for cervical cancer, in work-up radiological investigations, and in treatment delineation, planning, and delivery of radiation. FDG–PET is a valuable imaging tool in LN status definition for cervical cancer. Regarding treatment planning in radiotherapy, PET offers advantages in tumor delineation and defines the real impact of this imaging modality in radiation treatment planning and further treatment decision-making.

MRI remains an important diagnostic tool in pretreatment work-up and its role is obtaining exceptional results in tumor delineation for EBRT and in IGABT, providing higher spatial soft tissue resolution. The general tumoral description and extension depicted by MRI include a more accurate assessment of tumor size (due to multiplanar evaluation), which could reduce staging morbidity, and, therefore, superior results in terms of toxicity post-treatment.

IGRT and IGABT are promising technique tools that can achieve greater local control while reducing complication rates in LACC patients. These modalities are complementary and should be implemented in prospective trials with a move towards a more personalized line of action. Technology advances with superior imaging together with magnetic-resonance-guided radiation therapy and 3D printing in IGABT may hold the greatest potential to improve treatment and reduce potential side effects.

Although there is a possibility to introduce adaptive strategies with clinical benefit, several improvements in automated planning, quality assurance, and imaging quality need to be developed.

## Data Availability

Data are available upon request from the corresponding authors.

## Abbreviations

ADC	apparent diffusion coefficients
BT	brachytherapy
CBCT	cone beam computed tomography
CI	confidence interval
CRT	chemo-radiotherapy
CT	computed tomography
CTV	clinical target volume
DCE MRI	dynamic contrast-enhanced MRI
DW-MRI	diffusion-weighted MRI
DFS	disease free survival
DRR	digitally reconstructed radiograph
EBRT	external beam radiotherapy
EMBRACE	European study on MRI-guided brachytherapy in locally advanced cervical cancer
EQD2	biologically equivalent dose in 2 Gy fractions
FDG	fluorodeoxyglucose
FIGO	International Federation of Gynaecology and Obstetrics
GEC-	The Groupe Européen de Curiethérapie and the European society for ESTRO radiotherapy & oncology
GOG	Gynecologic Oncology Group
GTV	gross tumor volume
HDR	high dose rate
HR CTV	high risk clinical target volume
ICRU	International Comission on Radiation Units and Measurements
IGABT	image-guided adaptive brachytherapy
IGRT	image-guided radiotherapy
IMRT	intensity modulated radiation therapy
IR CTV	intermediate risk clinical target volume
ITV	internal target volume
LACC	locally advanced cervical cancer
LC	local control
LDR	low dose rate
LINAC	linear accelerator
LN	lymph node
MLC	multileaf collimator
MRI	magnetic resonance imaging
OARs	organs at risk
OS	overall survival
OTT	overall treatment time
PA	paraaortic
PDR	pulsed dose rate
PET	positron emission tomography
PTV	planning target volume
RTOG	Radiation Therapy Oncology Group
SIB	simultaneous integrated boost
SWOG	Southwest Oncology Group
T	Tesla
